# On the Relationship of Interoceptive Accuracy and Attention: A Controlled Study With Depressed Inpatients and a Healthy Cohort

**DOI:** 10.3389/fpsyg.2020.597488

**Published:** 2021-02-01

**Authors:** Dana Schultchen, Carolin Schneider, Götz Berberich, Michael Zaudig, Thorsten M. Erle, Olga Pollatos

**Affiliations:** ^1^Clinical and Health Psychology, Institute of Psychology and Education, Ulm University, Ulm, Germany; ^2^Psychosomatic Clinic Windach am Ammersee, Windach, Germany; ^3^Psychotherapeutisches Gesundheitszentrum und Medizinisches Versorgungszentrum am Goetheplatz, Munich, Germany; ^4^Department of Social Psychology, School of Social and Behavioral Sciences, Tilburg University, Tilburg, Netherlands

**Keywords:** depression, interoception, heartbeat perception, attention, psychosomatic

## Abstract

**Objective:**

Previous research has shown reduced interoceptive accuracy (IAcc) in depression. Attention deficit represents a key symptom of depression. Moreover, IAcc is positively correlated with attention. There is no study that investigates the effect of depression on IAcc and attention. The aim of this study is to examine the mediating effect of IAcc on depression and attention.

**Methods:**

Thirty-six depressed patients from the Psychosomatic Clinic in Windach were matched with 36 healthy controls according to age and sex and were assessed at Ulm University. All participants completed the Beck Depression Inventory-II, the heartbeat perception task to examine IAcc, and the d2 test assessing selective attention.

**Results:**

Depressed patients showed attention deficits—both for general visual attention and IAcc—compared to healthy controls. The mediation analyses revealed that the relationship between depression and attention is not mediated via IAcc. Furthermore, depression predicts IAcc and attention, but these effects are direct and largely unaffected by the respective other variable.

**Discussion:**

The results of the present study highlight both interoceptive as well as attention deficits in depressed patients. No clear mediation between these variables could be shown in this study. More elaborative research is needed to clarify whether different approaches to improve IAcc are effective for these deficits in depressed patients and could therefore be of importance as an additional aspect of therapy in depression.

## Introduction

Affecting over 300 million people (4.7%) worldwide, major depression is one of the most common mental diseases (World Health Organization [WHO], 2017). Moreover, due to the high number of affected people, it represents a severe public health burden (Greenberg and Birnbaum, 2005; Wittchen et al., 2011; Ferrari et al., 2013; World Health Organization [WHO], 2017). Depressed people experience impairments of different domains, including affective and cognitive symptoms (e.g., loss of interest, feelings of guilt, or concentration problems) as well as somatic distress. Moreover, depressive episodes are characterized by a loss of appetite, psychomotor retardation, or agitation (World Health Organization [WHO], 2009).

Typical problems in depressed patients such as decreased perception and regulations of emotion and stress as well as the perceptions of hunger and satiety are related to the concept of interoception, which is described as the ability to perceive and sense internal bodily signals (Vaitl, 1996; Cameron, 2001; Craig, 2002). Garfinkel et al. (2015) proposed a three-dimensional model of interoception, which includes interoceptive accuracy (IAcc), interoceptive sensibility (IS), and interoceptive awareness (IAw). IAcc is described as the perception of internal bodily signals and is objectively measured by the heartbeat perception task (Schandry, 1981). The second dimension, IS, denotes a subjective parameter and is thereby usually measured by self-rating questionnaires [e.g., Body Perception Questionnaire (BPQ); Porges, 1993, 2015, Multidimensional Assessment of Interoceptive Awareness (MAIA); Mehling et al., 2012), and confidence ratings directly after an IAcc task (Garfinkel et al., 2015). IAw as the third dimension represents the congruence of IAcc and IS, which is defined as the meta-cognitive level of interoceptive processes. In this article the focus is on IAcc, which represents the main dimension of interoception and is associated with IS as well as IAw.

One important association in the context of interoception is its relationship with emotion. This association is based on different emotion theories. James (1884) and Lange (1887) described in their James–Lange theory that emotions result from visceral and vascular changes; Schachter and Singer (1962) added that the interpretation of an arousal is important to form an emotion. In addition, Damasio’s (1994) somatic marker hypothesis exemplified the body-relatedness of emotional signals. All theories emphasize the visceral and somatic feedback to the brain, which in turn influences emotional behavior and facilitates decision-making processes (Damasio, 2000). Overall, several studies support the idea of these theories, showing a close relationship of interoception and emotions (Dunn et al., 2007; Herbert et al., 2011; Füstös et al., 2013). Findings mostly indicate that decreased IAcc is associated with higher alexithymia scores (Herbert et al., 2011; Shah et al., 2016; Murphy et al., 2018; Pollatos and Herbert, 2018; Zamariola et al., 2018), reduced arousal ratings (Dunn et al., 2010), and downregulation of affect (Füstös et al., 2013). Besides emotion regulation, different studies found an impaired IAcc in several clinical and subclinical samples (e.g., anorexia nervosa, fibromyalgia, somatoform disorder; Pollatos et al., 2008; Weiss et al., 2014; Fischer et al., 2016; Duschek et al., 2017).

Depression symptoms are also associated with problems in IAcc in non-clinical and clinical samples (Dunn et al., 2007; Pollatos et al., 2007, 2009; Furman et al., 2013). For instance, Pollatos et al. (2007, 2009) showed that higher depression scores are linked with an impaired IAcc in a non-clinical sample. Similarly, these findings could also be demonstrated for clinical samples: Dunn et al. (2007) and Furman et al. (2013) found that depressed patients had a significantly lower IAcc in comparison to healthy controls. It should be mentioned that Furman et al. (2013) investigated only women and that the findings in the study of Dunn et al. (2007) could not be supported among patients with severe depression. Moreover, reduced positive affectivity, impaired emotion regulation strategies, and changes in visceral feedback from the body were also found in previous studies (Silk et al., 2003; Joormann and Gotlib, 2010; Wiebking et al., 2010; Furman et al., 2013), which could explain the decreased IAcc.

Cognitive processes are another influencing factor on IAcc. The assumption that heightened attention to interoceptive signals is required to perceive them, is shown in two studies (Gregory et al., 2003; Matthias et al., 2009). Exemplarily, Matthias et al. (2009) found that participants with higher IAcc performed better in the d2 Test of Attention, which represents a better (visually selective) attention. Nonetheless, these results are only revealed by a correlational design of a healthy cohort. Beyond that, different neuroimaging studies showed an overlap between attention and different neural networks (e.g., insular cortex, prefrontal cortex, anterior cingulate), which also underlines the relationship between attentional performance and interoceptive sensation (Corbetta et al., 1991; Phan et al., 2002; Nebel et al., 2005). Based on the defined symptoms of the International Classification of Disease, tenth revision (ICD-10), attention and concentration problems are common symptoms in depressed patients (Paelecke-Habermann et al., 2005; McDermott and Ebmeier, 2009; World Health Organization [WHO], 2009). Even though specific findings on the relationship between IAcc in depressed patients and attention remain scarce, cognitive processes seem to have an impact on both IAcc and depression and would be of interest for further research.

So far, research shows that a higher IAcc, so called attention for bodily signals, has a higher visually selective attention (Matthias et al., 2009). Until now, there is no study that investigates the influence of depression in this field. Therefore, interoception and attention were investigated in a sample with depressed patients in comparison to matched healthy controls. Based on this, we hypothesize reduced IAcc in depressed patients in comparison to healthy controls. Similarly, we assume that depressed patients exhibit decreased attention (d2 test) compared to healthy controls. Moreover, we assume a negative relationship between depression and IAcc as well as the attention level. To expand the research question, it is important to bring all these variables together and to investigate whether the negative association between attention and depression is mediated by a reduced IAcc. To examine the interrelations between the concepts of depression, interoception, and attention, it is hypothesized that a possible relationship of depression to decreased attention is mediated by reduced interoceptive abilities.

## Materials and Methods

### Participants

All descriptive data are summarized in Table 1. The depressive sample comprised 36 patients (17 male) with a mean age of 41.19 years (*SD* = 11.13). All patients fulfilled the ICD-10 criteria for a major depression and were recruited from the Psychosomatic Clinic in Windach am Ammersee, Germany. Semi-structured diagnostic interviews according to ICD-10 criteria were used to determine the diagnosis of major depression. Testing procedures were conducted in the first or second week of inpatient therapy. Regarding former therapy experiences, 18 patients specified that they were in an ambulant setting, one took part in another inpatient setting, 15 had previously received therapy as inpatients and outpatients. Only two patients did not have any previous experience of a therapy setting. Furthermore, 22 patients were treated with antidepressants. Most of the patients (*n* = 23) had at least one comorbid psychiatric disorder, including personality disorder (*n* = 11), somatoform disorder (*n* = 6), and agoraphobia (*n* = 6).

**TABLE 1:** Descriptive variables of depressive patients and healthy controls.

	Depressive patients	Healthy controls	Test statistics	*p*
		
	*n* = 36	*n* = 36		
Age in years *M* (SD)	41.19 (11.13)	40.92 (11.51)	*t*(70) = 0.104	0.92
Gender (% male)	47%		χ^2^(1) = 0.000	1.00
Education			χ^2^(1) = 6.32	0.18
(1)	7	3		
(2)	14	8		
(3)	3	5		
(4)	12	19		
(5)	0	1		
BDI-II mean (SD)	22.81 (9.08)	4.94 (5.02)	*t*(70) = 10.32	≤0.001

**M*, mean; SD, standard deviation; *N*, number; % *m*, percentage of males; *BDI-II*, Beck Depression Inventory-II; Education: (1) secondary general school certificate, (2) intermediate school certificate, (3) entrance qualification for technical college, (4) entrance qualification for university, (5) other qualification.*

Depressed patients were matched according to age and gender to 36 healthy controls with a mean age of 40.92 years (*SD* = 11.51). Healthy controls were excluded if they reported any medication intake (except of contraceptives) or a current psychiatric/somatic disorder.

Table 1 shows that depressed patients scored significantly higher on the Beck’s Depression Inventory (BDI-II) than healthy controls. It should be noted that none of the healthy controls scored higher than 19 on the BDI-II, meaning no healthy control exhibited a critical score at the time of the study (Kendall et al., 1987). No significant differences were found regarding age, sex, and educational level (see Table 1).

At the end of the testing procedure, participants received 10 € for taking part in the study. The study was conducted in accordance with the Declaration of Helsinki. Ethical approval was obtained from the Institutional Review Board of Ulm University.

### Instruments

Participants had to answer questions regarding demographic information such as age, sex, and education. Moreover, they received a test battery with standard psychology questionnaires. For the results presented in this study, the BDI-II (Beck et al., 1996) is relevant to quantify depressive symptoms over the previous two weeks. This well-validated self-reported questionnaire consists of 21 items, rated on a scale from 0 (=*not at all*) to 3 (=*always*), resulting in score ranges between 0 and 63. Higher scores represent more severe levels of depression (Beck et al., 1996; Steer and Clark, 1997; Arnau et al., 2001). Reliability scores for Cronbach’s α differ between 0.84 ≤ α ≤ 0.94 for clinical and non-clinical samples (Steer and Clark, 1997; Steer et al., 1998; Arnau et al., 2001; Kühner et al., 2007).

Interoceptive accuracy was measured via the heartbeat perception task by Schandry (1981) to investigate the relationship between IAcc and depression. Consequently, we used this well-validated task characterized by a good test-retest reliability (Schandry, 1981; Knoll and Hodapp, 1992; Mussgay et al., 1999). For reliable measurements, it is important to provide standardized instructions for the heartbeat perception task, including the following different aspects: First, participants were instructed to count their heartbeat silently without taking their pulse or holding their breath. They were told to only focus on their heartbeat, and not on their breathing. If they wanted, participants could close their eyes. Second, they had to choose a relaxed sitting position. It was necessary for participants to stay calm and to not talk during the task. Third, the instructor provided information that the task would include four intervals (25, 35, 45, and 60 s, randomly presented) and that participants would have a break between the intervals to report their counted heartbeats verbally. A 10 s training interval was integrated to familiarize participants with the task. Importantly, participants were neither informed about the length of the different intervals, nor they did receive any feedback regarding their performance. The instructor gave a verbal start- and stop signal for each interval, demonstrating the beginning and the end of heartbeat counting.

For the heartbeat perception task, the IAcc score is of high importance. This score indicates the accordance between counted and recorded heartbeats. The BIOPAC MP35 heart rate monitor (sampling rate of 1,000 Hz) was used for recording the heartbeats. Analyses of cardiovascular signals were performed with the corresponding software AcqKnowledge (version 4.4). The final IAcc score was calculated using the following equation:

$$\text{IAcc score}=\;\frac{1}{4}\sum \left(1-\frac{\left(\left|\text{recorded heartbeats}-\text{counted heartbeats}\right|\right)}{\text{recorded heartbeats}}\right)\;$$

This score can vary between 0 and 1. Higher scores indicate better IAcc and consequently a small difference between recorded and counted heartbeats.

For the assessment of attention, the d2 Test of Attention (Brickenkamp, 1998) was used. This test is a standardized method to measure processing speed and performance quality to quantify visual attention and concentration. The test material consists of a sheet of paper with the letters *d* and *p* which are arranged in 14 rows, with 47 letters in each row. Above and/or below each letter, there are one to four dashes. To perform the test, participants were asked to cross out only *d*s with two dashes, regardless of whether the dashes were above and/or below the letter. Before the test, participants got the chance to practice the task in one line. The instructor gave verbal start and stop signals and instructed the participant to move on to the next line after 20 s on each line. To evaluate a participant’s performance, the total number of processed items and incorrect letters were counted. Incorrect answers were subtracted from the total number of processed items to find the score of correct answers.

### Procedure

All participants were informed about the study and provided written informed consent. The testing of the depressed patients took place in the Psychosomatic Clinic in Windach am Ammersee. Healthy participants performed the testing procedure in the laboratories of the Clinical and Health Psychology Department at Ulm University. Both settings were comparable (quiet room, testing situation). All participants first filled out the demographics and BDI-II questionnaires along with other questionnaires not reported here. Next, the heartbeat perception task took place. Afterward, participants had to perform the d2 Test of Attention. In total, each session lasted about 45 min.

### Data Analysis

All statistical analyses were conducted using the program Statistical Packages for Social Science (SPSS, version 24). A *p-*value less than 0.05 was considered as significant. In cases where the Levene-test indicated differences in variances, corrected values were used. To analyze differences between depressed patients and healthy controls with regard to IAcc and attention (d2 test), *t*-tests for independent samples were calculated. One-sided correlation analyses were then utilized to investigate the possible influence of depression on attention mediated by IAcc. Mediation analyses were carried out using the SPSS macro-script (Process; Model 4) provided by Hayes (Preacher and Hayes, 2004, Preacher and Hayes, 2008), which uses a bootstrapping resampling strategy in order to examine the significance of the model and the effect of the mediator. As described by Hayes, for indirect effects, 95% bias-corrected bootstrapped confidence intervals were performed using 5,000 repetitions. The hypothesized mediation model tested depression as the independent variable, attention as the dependent variables, and IAcc as mediator. Additionally, a second mediation model with attention as the mediator and IAcc as the dependent variable was computed to test the reverse mediation path. The total effect (sum of the direct and of the indirect effect), the direct effect (effect of the independent variable without the effect of the mediator), the indirect effect (i.e., the mediation), standardized coefficients, and significance levels were reported.

## Results

### Differences in Interoceptive Accuracy and Attention in Depressed Patients

**FIGURE 1:**
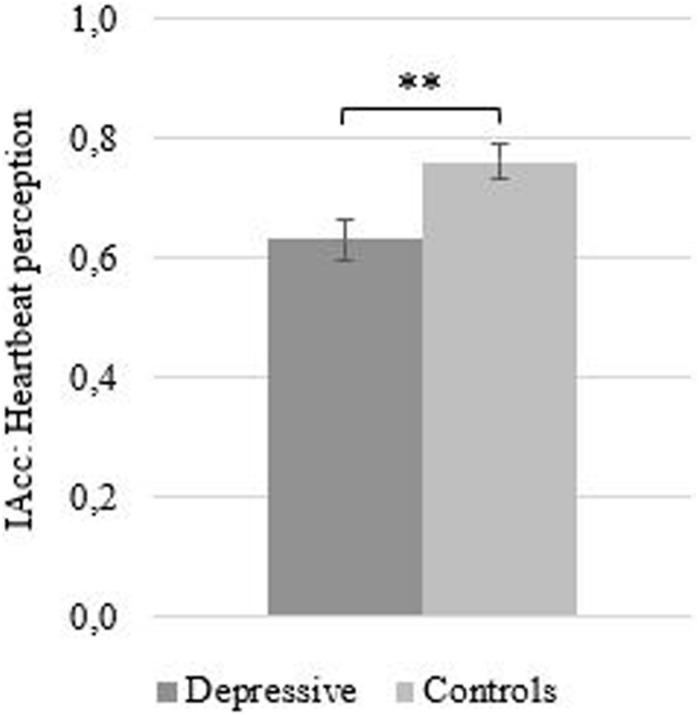
Mean (SE) interceptive accuracy as measured by the heartbeat perception task as a function of group. ^∗∗^*p* ≤ 0.01.

Compared to healthy controls, IAcc scores were lower in depressed patients [*M*_*Depr*_ = 0.62 (*SD* = 0.21); *M*_*Contr*_ = 0.77 (*SD* = 0.17)]. Results of the *t*-test for independent samples showed that IAcc scores differed significantly between depressed patients and healthy controls [*t*(70) = −3.23, *p* ≤ 0.01; see Figure 1]. Moreover, results showed that higher levels of depression are related with lower IAcc (*r* = −0.40, *p* ≤ 0.01).

**FIGURE 2:**
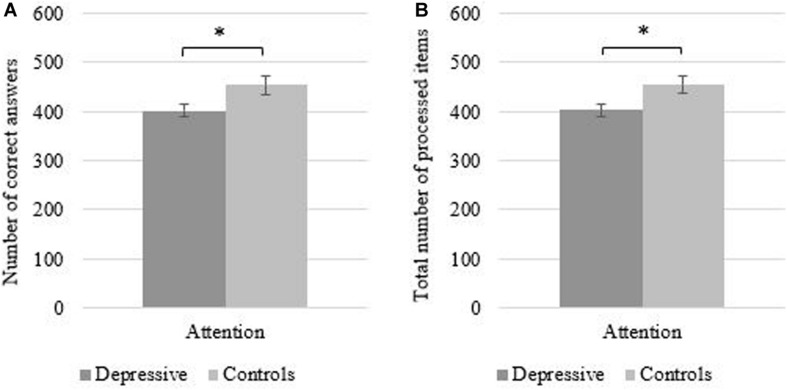
Mean (SE) total number of processed items **(A)** and mean correct answers **(B)** in the d2 test of attention as a function of group. ^∗^*p* ≤ 0.05.

Regarding attention, depressed patients gave significantly fewer correct answers than healthy controls [*t*(70) = −2.14, *p* ≤ 0.05; see Figure 2A] and also processed fewer items in total [*t*(70) = −2.14, *p* ≤ 0.05; see Figure 2B] on the d2 test. Thus, they exhibited deficits in two common indicators of attention within the d2 test. Due to the fact that the number of incorrect answers in depressed patients (*M* = 1.03, *SD* = 2.18) and controls (*M* = 0.72, *SD* = 1.56) were close to zero, no significant differences for the incorrect answers in both groups could be found [*t*(70) = 0.70, *p* = 0.49], which likely indicates a floor effect in the data. Therefore, in the following we focus our analysis on the more diagnostic metric of correct and total items completed. As analyses in this study with these two variables were almost identical, calculations for the following analyses were only presented with the total number of processed items. Across the entire sample, depression was significantly negatively correlated to the total number of processed items in the d2 test (*r* = −0.26, *p* ≤ 0.05) and correct answers (*r* = −0.26, *p* ≤ 0.05), suggesting that higher levels of depression predict lower attention scores. Lastly, no significant relationship between IAcc and attention (total number of processed items, *r* = 0.09, *p* = 0.22) emerged.

### Depression, Interoceptive Accuracy, and Attention

**FIGURE 3:**
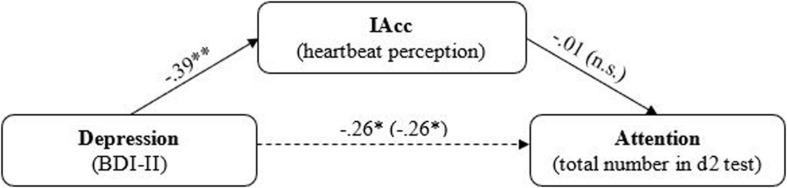
Mediation model for the effect of depressive on attention mediated by IAcc. Standardized regression coefficients are reported arrows. Regression coefficient from the regression analysis without mediator are depicted in parentheses. n.s., not significant. ^∗^*p* ≤ 0.05; ^∗∗^*p* ≤ 0.01.

To investigate the assumption that impaired IAcc mediates the relationship between depression and attention, a mediation analysis was performed. A representation of the findings is depicted in Figure 3.

The analyses conducted on the first model showed that depression significantly predicted IAcc (β = −0.39, *p* ≤ 0.001; see Figure 3). Similarly, depression also significantly predicted attention, both when controlling for the mediator (β = −0.26, *p* ≤ 0.05) and when performing a linear regression analysis independent of IAcc (β = −0.26, *p* ≤ 0.05). Thus, findings indicated a statistically significant total effect [β = −0.26, 95% CI (−0.486, −0.027)] and direct effect [β = −0.27, 95% *CI* (−0.513, −0.008)] of depression on attention, but no significant indirect effect of depression on attention, mediated via IAcc [β < 0.01, 95% CI (−0.111, 0.095)].

The reverse mediation test revealed that although depression significantly predicted attention (see above)—here in the role of the mediator—attention did not significantly predict IAcc [β = −0.01, 95% CI (−0.233, 0.214)], and therefore there was also no indirect effect of depression on IAcc, mediated via attention [β < 0.01, 95% CI (−0.058, −0.061)]. Thus, the results indicate that depression significantly and negatively predicts both IAcc and attention, but these effects are direct and largely unaffected by the respective other variable.

## Discussion

The purpose of the study was to investigate the relationship between depression, attention and IAcc. Therefore, depressed participants and healthy matched controls were examined through self-report questionnaires (BDI-II) and behavioral tasks (heartbeat perception task and d2 Test of Attention). Results indicated decreased IAcc and attention for depressed participants compared to healthy controls. Moreover, a negative relationship between depression and IAcc was found and could also be confirmed for depression and attention, but not for IAcc and attention. Mediation analyses revealed that IAcc did not mediate the negative relationship between depression on attention.

### Interoceptive Abilities in a Sample With Depressed Patients

It was hypothesized that depressive patients show decreased IAcc. In line with this assumption, depressive patients scored significantly lower than healthy controls on IAcc. Previous data from Furman et al. (2013) support our findings by the fact that participants with higher depression scores exhibited a lower performance in the heartbeat perception task. Additionally, results in our study indicated that higher depression scores are related with lower IAcc. Our findings add to the results of current research investigating the, thus far, inconsistent picture of the relationship of depression and interoceptive abilities (Dunn et al., 2007; Pollatos et al., 2009).

### Attention in a Sample With Depressed Patients

Our second main finding supports our hypothesis that depressed patients exhibit impaired attention capacity. Moreover, we found a negative correlation between depression and attention, which is reflected in the result that depression significantly predicted reduced attention. These findings are also supported by previous literature (Paelecke-Habermann et al., 2005; McDermott and Ebmeier, 2009) as well as in the ICD-10, where decreased concentration is listed as a key symptom (World Health Organization [WHO], 2009).

### The Relationship of Depression, Interoceptive Abilities, and Attention

Focusing on the last hypothesis, we combined all variables in a meditation analysis, assuming that the negative relationship between depression and attention is mediated by IAcc. Mediation analyses confirmed a negative relationship of depression to IAcc and a direct, negative effect of depression to attention. However, the indirect effect demonstrating the overall mediation did not reach significance. It could therefore not be shown that interoceptive abilities mediate the effect of depression to attention. A more detailed analysis revealed that the correlation coefficient of IAcc and attention was close to zero. This indicates almost no association between IAcc and attention in this study. This missing association of IAcc and attention is somewhat surprising and not in accordance with the research of Matthias et al. (2009). In contrast to the former study our sample consisted of patients with a significant decrease in attention as compared to the healthy controls and therefore other relevant variables such as level of depression or mood could be qualitative discrete additional factors that come into play. Nevertheless, descriptive data showed lower scores in IAcc and reduced attention scores in depressive patients as compared to healthy controls. Due to the novelty of our study approach, further research is needed to shed more light on the interrelations of depression, interoception, and attention.

### Strengths, Limitations, and Future Research

In summary, our study highlights the impact of bodily signal processing for basic aspects of depression, focusing on the intensity of depressive symptoms as well as basic aspects of attention. Until now, studies have exclusively investigated attention, and the relationship between IAcc and depression, separately. We have examined these variables and how they interact with each other, therefore showing a more concise picture as compared to former studies.

There are several limitations of the current study relating to sample characteristics and measurements, which should be considered in future research. For example, with a larger sample size, it could be useful to differentiate between the severity of depression, comparable to the study of Dunn et al. (2007). An important avenue for future research could also be investigating the influence antidepressant and comorbidities have on interoceptive abilities. Moreover, additional examinations of the heartbeat evoked potential (HEP) could be of interest. Montoya et al. (1993) examined HEP’s in two different conditions of attention (directed attention versus distraction) and found significant differences in central HEP amplitudes. Finally, it could be interesting for future research to measure depressive symptoms and interoception across time to find out more about the evolution of depressive symptoms and its association with IAcc. Following the model of Murphy et al. (2019), a daily assessment of interoceptive abilities in a depressed sample could be also interesting.

## Conclusion

In summary, results showed that depressed participants have deficits in both interoceptive abilities as well as in basic attention processes. It is an interesting aspect that this stands in contrast to our hypotheses that these deficits seem to be barely connected. This leads to the open question on whether there is a favorable order in therapeutic programs aiming at an improvement of interoceptive abilities or at the improvement of attention deficits. This is a relevant aspect for clinical practice as research so far has shown that through different methods, interoceptive abilities can be increased, including mindfulness meditation (Farb et al., 2013), body scans (Fischer et al., 2017), contemplative mental training (Bornemann et al., 2014; Bornemann and Singer, 2017), and self-focused training (Ainley et al., 2012). Whether this is also true for depressed patients or whether this effect might be more pronounced when attention processes are targeted first, has thus far not been investigated and requires more future research for clarification. Future research should re-examine this model and a possible mediation effect with a larger sample size, including possible influencing factors such as severity of depression, medication, and comorbidities.

## Data Availability Statement

The raw data supporting the conclusions of this article will be made available by the authors, without undue reservation.

## Ethics Statement

The study was conducted in accordance with the Declaration of Helsinki. Ethical approval was obtained from the Institutional Review Board of Ulm University. The patients/participants provided their written informed consent to participate in this study.

## Author Contributions

DS, GB, MZ, and OP designed and conceived the study. DS and CS collected the data and wrote the first draft of the manuscript. DS, CS, TE, and OP conducted the statistical analyses. All authors reviewed, edited, and approved the final manuscript.

## Conflict of Interest

The authors declare that the research was conducted in the absence of any commercial or financial relationships that could be construed as a potential conflict of interest.
